# Detection of Bacterial Endospores in Soil by Terbium Fluorescence

**DOI:** 10.1155/2011/435281

**Published:** 2011-07-07

**Authors:** Andrea Brandes Ammann, Linda Kölle, Helmut Brandl

**Affiliations:** ^1^Institute of Evolutionary Biology and Environmental Studies (IEU), University of Zurich, Winterthurerstraße 190, CH-8057 Zurich, Switzerland; ^2^Environmental Biotechnology, Zurich University of Applied Sciences (ZHAW), Grüental, CH-8820 Wädenswil, Switzerland

## Abstract

Spore formation is a survival mechanism of microorganisms when facing unfavorable environmental conditions resulting in “dormant” states. We investigated the occurrence of bacterial endospores in soils from various locations including grasslands (pasture, meadow), allotment gardens, and forests, as well as fluvial sediments. Bacterial spores are characterized by their high content of dipicolinic acid (DPA). In the presence of terbium, DPA forms a complex showing a distinctive photoluminescence spectrum. DPA was released from soil by microwaving or autoclaving. The addition of aluminium chloride reduced signal quenching by interfering compounds such as phosphate. The highest spore content (up to 10^9^ spores per gram of dry soil) was found in grassland soils. Spore content is related to soil type, to soil depth, and to soil carbon-to-nitrogen ratio. Our study might provide a basis for the detection of “hot spots” of bacterial spores in soil.

## 1. Introduction

The formation of spores is a survival mechanism of microorganisms when exposed to unfavorable environmental conditions (e.g., heavy metal stress, nutrient limitations) leading to a “dormant” or “resting” growth state [1, 2]. A variety of bacteria identified in diverse habitats including soil is able to form endospores. These physiological groups include aerobic heterotrophs (e.g., *Bacillus, Paenibacillus, Brevibacillus, Geobacillus, Thermoactinomyces, *and* Sporolactobacillus*), anaerobes (*Clostridium*, *Anaerobacter, *and* Desulfotomaculum*), microaerophiles (*Sporolactobacillus*), halophiles (*Sporohalobacter*), and phototrophs (*Heliobacterium*, *Heliophilum*) [3, 4]. Bacterial spores are characterized by a series of unique chemical features which can facilitate their identification in natural environments. Besides the high content of minerals (particularly calcium), spores contain high amounts of dipicolinic acid, DPA [5]. DPA is uniquely found in bacterial spores in amounts of up to 25% of the spore dry weight and depends on the bacterial species [6, 7]. In solution, a complex is formed in the presence of terbium which shows a very strong and distinctive fluorescence spectrum [8]. Originally, DPA was used to detect very low concentrations of terbium (III) [9]. On this basis, methods for the detection of bacterial endospores have been developed [10–13]: by the addition of terbium, the DPA content was determined.

However, terbium-DPA fluorescence might be interfered by a series of compounds, especially when DPA has to be determined in complex samples such as sediments or soils. It has been reported that the presence of phosphorus compounds (especially *ortho*-phosphate) reduced terbium fluorescence by as much as 98% [14]. The addition of aluminium compounds (especially aluminium chloride, AlCl_3_), however, ameliorated the interference caused by the quenching substances [14]. From a series of organic compounds (benzoate, tryptophan, tyrosine, phenylalanine, glucose, malate, riboflavin, NAD, and tryptone) only the latter two (especially tryptone) reduced fluorescence significantly. Carbohydrates (e.g., starch, dextrine) were reported not to interfere with the terbium fluorescence [15]. Inorganic compounds such as calcium carbonate, sodium chloride, potassium chloride, ammonium sulphate, ammonium nitrate, and sodium nitrate did not lead to a reduction of the fluorescence, but only dipotassium phosphate did [16].

The aim of this study was to adopt the fluorescence-based method to determine the spore content in soils sampled from various locations. In particular, we were interested in the differentiation between different types of soil such as grasslands (pasture, meadow), allotment gardens, and forests, as well as fluvial sediments, the relationship of soil parameters (carbon-to-nitrogen ratio) on the occurrence of bacterial spores, and the distribution of spores in relation to sampling depth.

## 2. Materials and Methods

### 2.1. Bacterial Spores

Different *Bacillus* species (*B. megaterium, B. subtilis*) were cultivated in liquid medium containing (in g/l): glucose (3.6), ammonium chloride (2.5), magnesium sulfate (0.2), calcium chloride (0.07), iron sulfate (0.01), EDTA (0.01), potassium dihydrogen phosphate (0.6), dipotassium hydrogen phosphate (0.9), and yeast extract (1.0). Initial pH was adjusted to 7.0. Erlenmeyer flasks (250 ml) containing 100 ml of growth medium were inoculated and incubated for 10 to 15 days (150 rpm, 30°C). To initiate and stimulate sporulation, bacteria were subsequently transferred to a sporulation medium (identical composition, but without glucose and less ammonium chloride [only 1 g/l]). After additional 30 days of incubation—until vegetative cells were not present anymore after inspection by microscopy—spores were harvested by centrifugation, immediately frozen in liquid nitrogen followed by lyophilization.

### 2.2. Soil Samples

Samples from different locations were collected using a stainless steel soil corer (15 mm in diameter), which was sterilized before each sampling. Cores with a maximum length of 25 cm were obtained, cut in sections of 5 cm, transferred to sterile screw cap Falcon tubes (20 ml), and stored on dry ice. After return to the laboratory, samples were immediately lyophylized or stored at −80°C until further processing.

Sampling sites were located in the surroundings of Zurich (Switzerland): grassland soil, meadow (municipalities of Männedorf; Uerikon; Stäfa; and Dübendorf), allotment garden (University of Zurich, Irchel campus), pasture (University of Zurich, Irchel campus; municipality of Wädenswil), forest soil (municipality of Stäfa), and aquatic sediments (river Glatt in Dübendorf).

Lyophilized aliquots of approximately 1 g were transferred to an Eppendorf micro test tube (2 ml) and ground (by adding a 6 mm glass bead) in TissueLyser (Retsch, Haan, Germany) for 5 × 1 min. Elemental composition (carbon, hydrogen, nitrogen) of soil was performed with a CHN-932 elemental analyzer (Leco Corp., St. Joseph, Minn, USA). Approximately 10 mg of powdered soil was used for analysis. Composition (in % of dry soil) varied between 2.2 and 15.4, 0.2 and 1.4, and 0.2 and 2.0 for total carbon, total hydrogen, and total nitrogen, respectively. Phosphate in aqueous soil extracts (250 mg soil in 5 ml sodium acetate buffer; 0.2 M, pH 5) was determined using commercially available kits (LCK 348 and 349; Hach Lange AG, Hegnau, Switzerland).

### 2.3. Release of DPA from Spores

10 mg of dry spore powder was resuspended in 10 ml sodium acetate buffer (0.2 M, pH 5). Spores were counted under the microscope using a Neubauer counting chamber. Soil samples were thawed and 50 mg were suspended in 0.9 ml sodium acetate buffer and 0.1 ml aluminium chloride (AlCl_3_, 0.5 M). Optimal volumetric amount and concentration of aluminium chloride was determined in preliminary experiments. Samples were microwaved (Berghof Microwave Digester MWS-1, with built-in *in situ* infrared temperature control) in Teflon TFM screw cap digestion vessels. Temperature and power were set to 140°C and approximately 680 W (80%), respectively. Alternatively, DPA was released from spores by autoclaving the samples in screw cap glass test tubes for 15 minutes at 121°C. The presence of spores after microwaving and autoclaving was determined by microscopy. The identical DPA extraction protocol was applied for soil samples. However, microscopy was not possible due to the presence of mineral particles interfering the observation.

### 2.4. Fluorescence Measurement

After cooling for 30 minutes, 100 *μ*l of the spore suspensions were mixed with 100 *μ*l terbium chloride solution (TbCl_3_, 30 *μ*M) in white 96-well microtiter plates (in 8 replicates). Fluorescence was immediately measured using a plate reader (SpectraMax M2, Bucher Biotec, Basel, Switzerland) with the following settings: time-resolved fluorescence (delay 50 *μ*s, interval 1200 *μ*s) at an excitation wavelength of 272 nm, emission wavelength of 545 nm, and 10 endpoint readings per sample at 30°C. The number of spores in the soil samples was determined using standard addition method with spores of *B. subtilis *[17]. Spore content was expressed as equivalents of *B. subtilis*.

## 3. Results and Discussion

**Figure 1:**
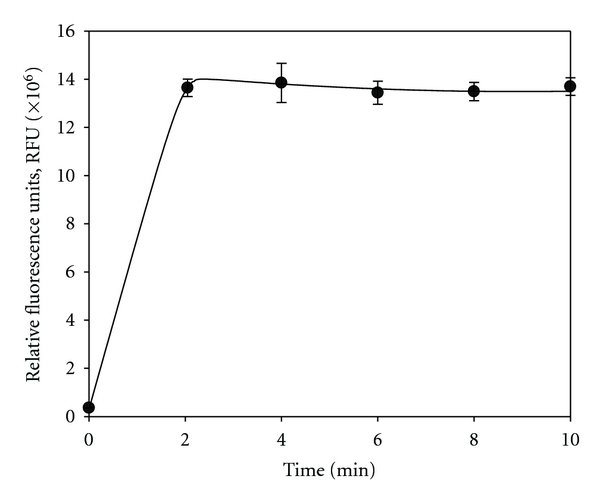
Release of dipicolinic acid (DPA) from a bacterial spore suspension of *B. megaterium* in relation to the duration of the microwave treatment. Points represent mean values of 8 replicates.

**Figure 2:**
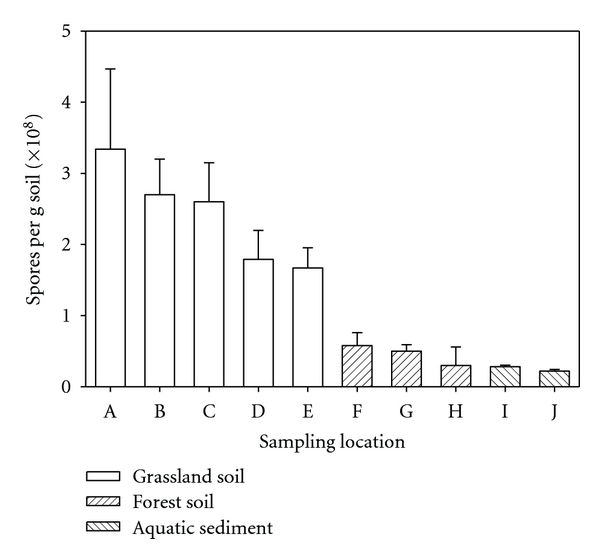
Spore content (expressed as equivalents of *B. subtilis*) of soil from ten different locations. A: grassland soil (municipality of Männedorf, site 1); B: grassland soil (municipality of Männedorf, site 2); C: grassland soil (municipality of Männedorf, site 3); D: grassland soil (municipality of Uerikon); E: grassland soil (municipality of Dübendorf); F: forest soil (municipality of Stäfa, site 1); G: forest soil (municipality of Stäfa, site 2); H: forest soil (municipality of Stäfa, site 3); I: aquatic sediment (river Glatt in Dübendorf, site 1); J: aquatic sediment (river Glatt in Dübendorf, site 2). Bars represent mean values of triplicates. Carbon, hydrogen, and nitrogen content (in % of dry soil) was for grassland soil 6.9 ± 0.6, 0.98 ± 0.07, and 0.54 ± 0.03; for forest soil 4.7 ± 2.3, 0.84 ± 0.29, and 0.32 ± 0.12; for aquatic sediments 6.5 ± 0.4, 0.36 ± 0.09, and 0.11 ± 0.03, respectively.

Microwave treatment of spore suspensions and soil samples led to a fast release of DPA (Figure 1). Within two minutes, maximum release was obtained. Increased treatment time did not improve DPA mobilization. Bacterial spore content was related to soil type (Figure 2). Highest spore numbers up to 4  × 10^8^ spores per gram dry soil were found in agriculturally used land (meadow, pasture), less in forest soil. Fluvial sediments showed lowest spore numbers. The interference of different compounds present in soil (e.g., phosphate) might lead to quenching of the fluorescence signal. This drawback has been overcome by the addition of aluminium chloride as already shown for the determination of bacterial spores in aquatic sediments [18]. Concentration of *ortho*-phosphate in soil extracts (22.5 *μ*M) was reduced by the addition of aluminium chloride to concentrations below the detection limit (<1.2 *μ*M). Concomitantly, a de-colorization of the extract was observed suggesting the removal of humic acids which have also the potential to form complexes with terbium and quench the fluorescence signal [17]. The method based on terbium fluorescence for the detection of total numbers of bacterial endospores in soils is fast and easy.

**Figure 3:**
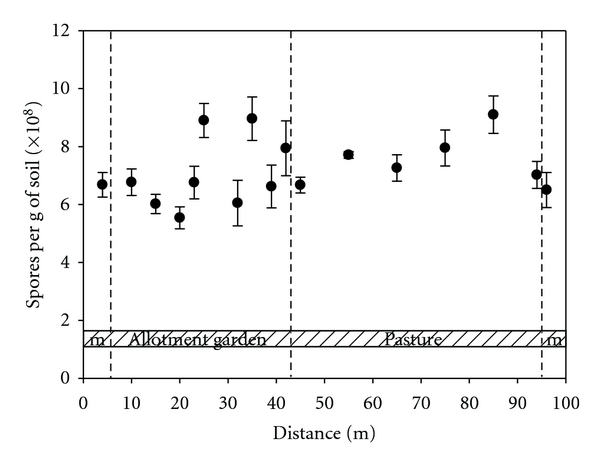
Transect of 100 m through a field showing different land use management: unused meadow (m), allotment garden, and pasture. Bacterial spore content is expressed as equivalents of *B. subtilis*. Data represent mean values of triplicates. Carbon, hydrogen, and nitrogen content (in % of dry soil) was 4.5 ± 0.6, 0.7 ± 0.1, and 0.3 ± 0.1, respectively.

A transect (approximately 100 m in length) through a grass field with different land use management (unused meadow, allotment garden, and pasture) gave spore numbers in the range of 5 to 9 × 10^8^ spores per gram of dry soil (Figure 3). Spore counts were not related to the type of land use: in allotment garden soil, counts were not significantly different from soil samples taken from a pasture (*P* = 0.423; *t*-test). Regarding the different sampling sites, our results show that grassland soils (meadow, allotment garden, and pasture) contains much more bacterial spores than forest soils and fluvial sediments.

**Figure 4:**
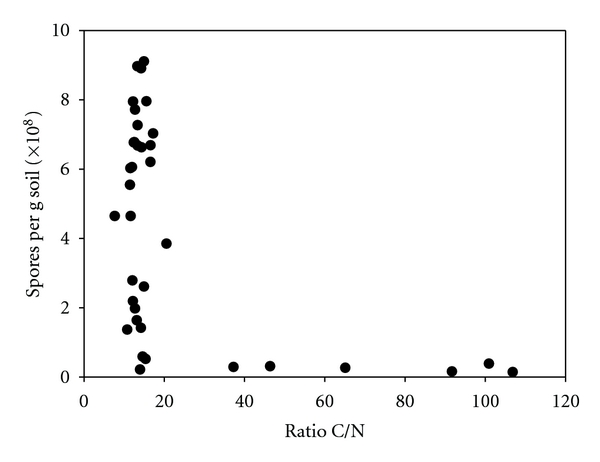
Spore number (expressed as equivalents of *B. subtilis*) as function of soil carbon-to-nitrogen ratio.

Spore content was related to the carbon-to-nitrogen ratio (Figure 4). At C/N ratios >20 only low spore counts (0.5 × 10^8^ spores per gram of dry soil) were detected as compared to C/N ratios <20. It has been demonstrated in pure cultures of *Bacillus thuringiensis* in a stirred bioreactor that low carbon-to-nitrogen ratios of 4 : 1 resulted in high spore counts [19]. In contrast however, spore formation in *Streptomyces coelicolor* was stimulated under nitrogen-limiting conditions [20]. In particular, C/N ratios between 50 and 100 promoted sporulation, whereas C/N rations <40 did not allow spore formation. Our results showed that in soils with extremely high C/N ratios, spore content was low. The importance of C/N ratio was stressed by Gao and coworkers regarding the sporulation of fungi, although fungal spores do not contain DPA [21]. A carbon-to-nitrogen (C/N) ratio of 20 stimulated spore formation by fungi such as *Penicillium camembertii* [22]. The fungus *Colletotrichum coccodes* produced highest spore counts at a C/N ratio of 5 to 10, whereas at a ratio of 40, spore formation was significantly lower [23]. Similarly, in *Plectosporium tabacinum* optimal spore formation was found when C/N rations were between 5 and 10 [24]. The distribution of spores in marine sediments (determined as DPA) showed only a low correlation with the content of total organic carbon and varied with the sediment type [18]. Highest numbers have been found in organic-rich black sediments, lowest number in sandy sediments.

It was hypothesized from anthrax outbreaks, that the high numbers of *Bacillus* spores might be related to soils rich in organic matter that is, to a high C/N ratio [25]. These soil environmental conditions are suggested to support the presence and viability of *B. anthracis* spores [26]. However, we could not confirm this hypothesis.

**Figure 5:**
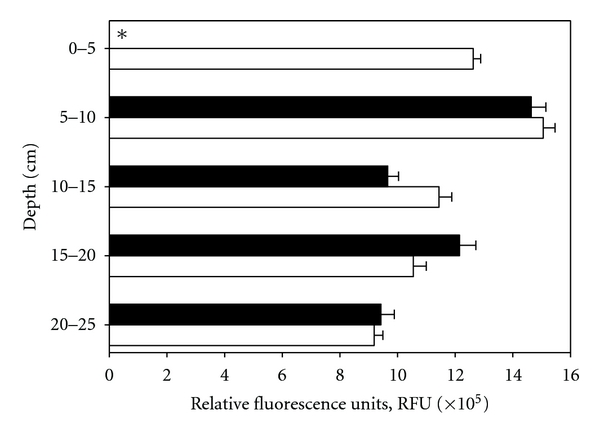
Depth profiles (0 to 25 cm) of bacterial spore content (expressed as equivalents of *B. subtilis*) in soil from a pasture. Comparison between autoclaving (open bars) and microwaving (solid bars) in releasing dipicolinic acid (DPA). Bars represent mean values of triplicate samples. *Sample was lost during filtration step.

Depth distribution of spores from an area currently used as allotment garden (cultivation of flowers and vegetables) showed highest numbers in a horizon of 5 to 10 cm (Figure 5). The two methods evaluated (microwaving, autoclaving) for the mobilization of DPA from bacterial spores gave similar results. However, microwaving was less time-consuming, whereas autoclaving allowed faster throughput of samples.

## 4. Conclusions

In summary, microwave treatment of soil samples followed by the measurement of fluorescence after addition of terbium proved to be a fast and easy method to assess the content of bacterial spores. Our study might provide a basis for the detection of “hot spots” of endospores in soil.
